# Primary placement technique of jejunostomy using the entristar™ skin-level gastrostomy tube in patients with esophageal cancer

**DOI:** 10.1186/1471-230X-11-8

**Published:** 2011-01-31

**Authors:** Yasushi Rino, Norio Yukawa, Hitoshi Murakami, Tsutomu Sato, Ken Takata, Tsutomu Hayashi, Takashi Oshima, Nobuyuki Wada, Munetaka Masuda, Toshio Imada

**Affiliations:** 1 Departments of Surgery, Yokohama City University, School of Medicine 3-9, Fukuura, Kanazawa-ku, Yokohama city, 236-0004, Japan; 2 Gastroenterological Center, Yokohama City University Medical Center, 4-57, Urafune-cho, Minami-ku, Yokohama city, 232-0024, Japan

## Abstract

**Background:**

We developed a skin-level jejunostomy tube (SLJT) procedure for patients undergoing esophagectomy using a skin-level gastrostomy tube (G-tube) (Entristar™; Tyco Healthcare, Mansfield, Mass), in order to improve their nutrition status and quality of life (QOL). We describe the procedure and the adverse effects of SLJT in patients with esophageal cancer (EC).

**Methods:**

Over a 24-month period (March 2008 to March 2010), there were 16 patients (mean age: 61.8 years; age range: 49-75 years; 15 men, 1 woman) who had Stage II or III EC. Primary jejunostomy was performed under general anesthesia during esophagectomy. The technical success and the immediate and delayed complications of the procedure were recorded.

**Jejunostomy techniques:**

SLJT placement using the G-tube (20Fr) was performed 20 cm from the Treitz ligament on the side opposing the jejunal mesenterium. The internal retention bolster was exteriorized through an incision in the abdominal wall. A single purse string suture using a 4-0 absorbable suture was performed. The internal retention bolster was then inserted into the jejunal lumen via the small incision. The intestine adjacent to the tube was anchored to the peritoneum using a single stitch.

**Results:**

The SLJT was successfully inserted in all 16 patients. No early complications were documented. Follow-up for a median of 107 days (range, 26-320 days) revealed leakage to the skin in four patients, including superficial wound infections in two patients. There were no cases of obstruction of the tube or procedure-related death.

**Conclusions:**

This SLJT placement technique using the G-tube is a safe procedure in patients with EC and allows the creation of a long-term feeding jejunostomy.

## Background

Jejunostomy has been widely adopted as an adjunct surgical procedure for esophageal cancer or as a route for postoperative nutrition administration. Since the enteral route is considered more physiologic, it is often said that enteral nutrition is safer and more efficacious than the parenteral route[1]. In complicated esophageal surgery and patients in a debilitated state, jejunostomy has become an accepted method of dealing with anticipated anastomotic malfunction, pneumonia, or recurrent nerve palsy. The use of a jejunostomy tube was first described in 1891 by Witzel[2]. Liffman and Randall reported the jejunostomy placement of a small plastic catheter with a Witzel tunnel[3]. Sano *et al*. reported a simpler jejunostomy technique using a Bard Button without the Witzel tunnel[4]. Most of the direct jejunostomy techniques require the construction of a self-closing valve and splint by suture plication of the jejunal serosa over a tube. The plication is used to prevent the occurrence of jejunal fistula. The jejunum is then sutured to the anterior parietal peritoneum to avoid intraperitoneal dislodgement of the tube. Due to the technical steps required for a standard jejunostomy, most surgeons resist using this procedure as a routine adjunctive measure. In addition, potential complications of the standard jejunostomy include possible residual small bowel fistula, the appearance of extensive adhesions around the parietal attachments of the jejunostomy tube, and partial obstruction of the jejunal lumen. Thus, we developed a new method of skin-level jejunostomy tube (SLJT) insertion using a skin-level gastrostomy tube (Entristar™; Tyco Healthcare, Mansfield, Mass) for patients undergoing esophagectomy, in order to improve their nutrition status and quality of life (QOL). Jejunostomy is performed using the Witzel tunnel at our institute. We selected the G-tube for jejunostomy due to our familiarity with its use in gastrostomy. The Entristar™skin-level gastrostomy tube (G-tube) is a short 20 Fr diameter tube with internal and external retention bolsters to secure its position and minimize movement. The feeding and/or decompression ports are fitted with an antireflux valve that may be closed with a cap when it is not in use. As with all skin-level devices, the Entristar™device is normally inserted as a replacement tube, when a mature tract has already been established during a previous percutaneous endoscopic gastrostomy (PEG) procedure. The technique described in this report is simpler than the standard jejunostomy. The rapidity with which it can be introduced, and the multiple benefits derived from its use, suggest its adoption for simple, as well as complicated, surgical problems.

The purpose of the present study was to retrospectively review our experience with the surgical method of primary insertion of the G-tube in patients with esophageal cancer (EC). We describe the procedure and SLJT complications in patients.

## Methods

Over a 24-month period (March 2008 through March 2010), there were 16 patients (mean age: 61.8 years; age range: 49-75 years; 15 men, 1 women) who had Stage II or III EC. Informed consent was obtained prior to placement of the jejunostomy tube. The SLJT placement was performed under general anesthesia during esophagectomy with laparotomy. The technical success and any immediate or delayed complications of the procedure were recorded.

This study was approved by the ethics committee of the Yokohama City University, School of Medicine and was conducted according to the Declaration of Helsinki. Informed consent was obtained from each patient prior to enrollment in the study.

## SLJT procedure

### 1: Jejunostomy techniques of using G-tube (20Fr)

**Figure 1:**
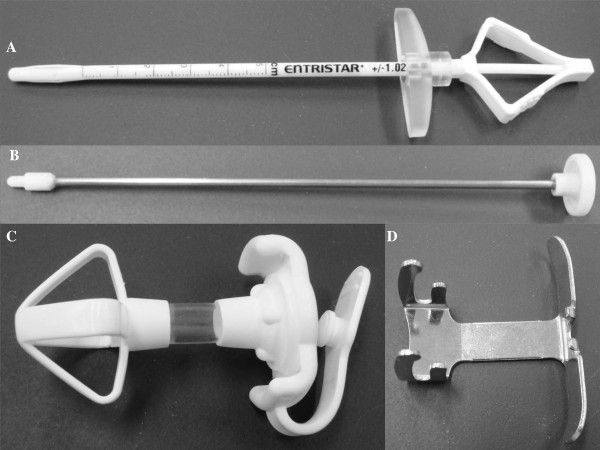
**Skin-level gastrostomy tube (Entristar; Tyco Healthcare, Mansfield, Mass)**. **A) **The Measuring device. **B) **The Obturator. **C) **Gastrostomy tube comprised of external and internal retention bolisters. **D) **The Gripstar.

We used a G-tube (20Fr) (Entristar; Tyco Healthcare, Mansfield, Mass) for the SLJT. The G-tube consisted of external and internal retention bolisters (Figure 1).

**Figure 2:**
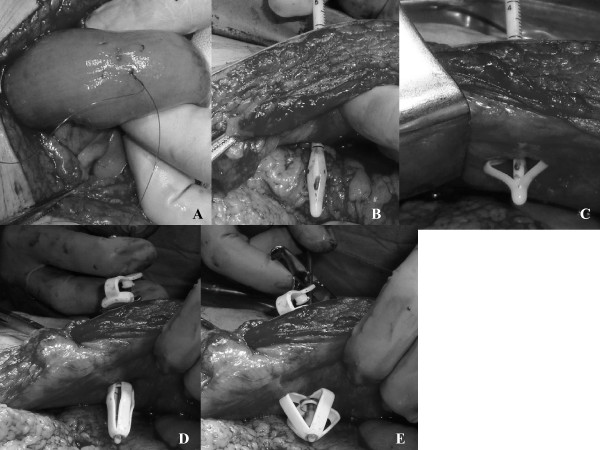
**Jejunostomy technique 1**. **A) **A purse string suture of the jejunostomy tube using a 4-0 absorbable suture. **B) **Insert the Stoma Measuring Device through the abdominal wall into the abdominal cavity. **C) **Measure the abdominal wall thickness. **D) **Use the finger grips on the GripStar Device for retention during obturation and insertion. **E) **Insert the obturated G-Tube into the stoma tract until the internal retention portion can be seen perfectly in the abdominal cavity.

During the procedure, the patient is placed in a supine position. We perform the jejunostomy during the abdominal procedure at the end of the esophageal cancer operation. We perform the jejunostomy 20 cm from the Treitz ligament on the side opposing the jejunal mesenterium. A single purse string jejunostomy stitch is made using a 4-0 absorbable suture (Figure 2.A). The stitch length is about 10 mm and four stitches are performed in a square shape around the jejunostomy site.

A skin incision, about 8 mm in length, is made at the left upper quarter of the abdomen. Forceps are then used to pierce the abdominal wall and the Stoma Measuring Device is inserted through the abdominal wall into the abdominal cavity (Figure 2.B). Based on the measurements obtained, a G-tube length is selected that allows for forward and backward-movement of the Entristar™device (Figure 2.C). This could help reduce complications as a consequence of continual or excessive pressure to either the jejunal mucosa or the skin.

We open the G-tube tethered cap, load the GripStar Insertion/Removal Device by sliding the lower curved prongs of the GripStar device beneath the external retention portion of the G-Tube, and use the finger grips on the GripStar Device for retention during obturation and insertion (Figure 2.D).

The obturator is inserted into the G-tube and we do not apply lubricating jelly. We obturate until moderate resistance is felt to reduce the diameter of the internal retention bolster. The obturated G-Tube is then inserted into the stoma tract until the internal retention portion can be seen perfectly in the abdominal cavity (Figure 2.E). If excessive resistance is encountered during insertion, the procedure is stopped and the tract is dilated.

**Figure 3:**
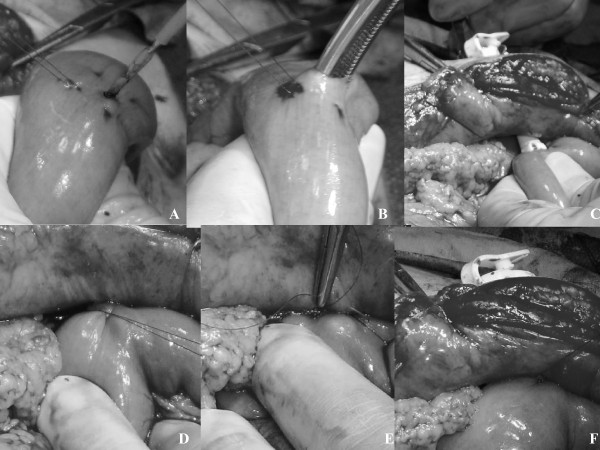
**Jejunostomy technique 2**. **A) **The center of the purse string suture of the jejunum is cut using an electronic knife. **B) **Pierce using forceps. **C) **Use obturator and insert the internal retention bolster into the jejunal lumen through the small incision. **D) **Purse string suture thread is tied tightly to the tube. **E) **The intestine adjacent to the tube is anchored to the peritoneum. **F) **Proper placement of the G-Tube after operation.

The center of the purse string suture of the jejunum is cut using an electronic knife (Figure 3.A) and pierced using forceps (Figure 3.B). We reduce the diameter of the internal retention bolster using the obturator and insert the internal retention bolster into the jejunal lumen via the small incision (Figure 3.C). A purse string suture is tied tightly to the tube (Figure 3.D). The intestine adjacent to tube is anchored to the peritoneum by a single stitch (Figure 3.E).

Proper placement of the G-Tube should be confirmed through the injection of 20 ml of air (Figure 3.F). Feedings should commence only after proper placement and patency have been confirmed.

A step-up spacer is placed beneath the external portion of the G-Tube.

The entire procedure requires less than five minutes to perform, and it adds very little time or trauma to the overall surgical procedure nor does it require significant intestinal manipulation.

### 2: How to use SLJT

The contrast medium, 20 ml Gastrografin, is administered through the SLJT to confirm that there is no leakage or obstruction on the first day after the operation. Liquefied standard diets can be subsequently administered via the SLJT. The starting dose of the liquefied standard diet is 20 ml/hr and is increased in 10 ml/hr daily increments to a maximum dose of 60 ml/hr.

### 3: Patient follow-up

Complications were characterized and were recorded as early (those occurring <24 hours after the procedure) or late (those occurring ≥24 hours after the procedure). Those complications arising after the procedure were further defined as minor (abdominal pain, wound infection, fever, peristomal leakage, dislocation, tract disruption, and catheter dislocation or fracture) or major (hemorrhage requiring blood transfusion, pneumoperitoneum, peritonitis, aspiration, and any complication of the tube insertion requiring radiologic intervention or surgery). Patient follow-up was assessed monthly, by the surgeons in all cases, to document subsequent complications. The occurrence of death was recorded and assessed to determine whether it had any relationship with the jejunostomy procedure. The tubes were removed at varying times, post-insertion, from 50 to 315 days, in 7 out of 16 cases. The needle tract was closed immediately without suture closure.

## Results

The results are summarized in the Table 1. The patients were followed up until removal of the G-tube for 26-320 days (median: 107 days) after tube insertion. The duration of administration of the liquefied diet was 7-319 days (median, 23.5 days) after tube insertion. In nine out of 16 SLJT cases, the tubes were not removed. SLJT placement was performed successfully in all 16 patients. There were no procedural failures as a consequence of the tube insertion technique; all tubes were correctly positioned. There were no early complications; i.e., no obstruction of the jejunal lumen and no tube failure as a result of tube fracture, displacement, or dislodgement. There were 4 cases of peristomal leakage, 1 case of dermatitis and 1 case of peristomal ulceration as late complications in 4 patients. No tube-related death or long-term major morbidity occurred as a consequence of the SLJT procedure.

**Table 1:** Clinical and Outcome Data for Patients with Esophageal Cancer Operation Who Underwent Entristar Gastrostomy Feeding Tube Placement

Patient No./Age(y)/Gender	Tube removal yes/no	Early (<24 b) Complications	Late (≥ 24 h) Complications	Duration (days)/follow-up until removal	Duration (days)/nutrient administration
1/49/M	yes	-	-	176	14
2/51/M	yes	-	-	106	12
3/69/M	no	-	-	192	85
4/60/M	yes	-	-	87	32
5/63/M	yes	-	-	101	9
6/64/M	no	-	peristomal leakage	201	200
7/66/M	yes	-	-	51	15
8/75/M	yes	-	-	108	69
9/61/M	no	-	ulceration, peristomal leakage	112	111
10/57/F	yes	-	dermatitis, peristomal leakage	319	105
11/57/M	no	-	peristomal leakage	320	319
12/63/M	no	-	-	114	112
13/66/M	no	-	-	26	9
14/61/M	no	-	-	65	7
15/67/M	no	-	-	74	13
16/59/M	no	-	-	55	9
					
			median	107	23.5

## Discussion

Enteral feeding via insertion of a PEG tube is standard procedure in many institutions and is the recommended procedure for long-term maintenance of good nutrition in patients with disorders that involve severe dysphagia and generalized weakness or immobility. Esophagectomy results in reduction of food intake, leading to malnourishment and weight loss. It is therefore important that enteral feeding is established at an early stage, in order to minimize further malnutrition, and jejunostomy is now regarded as the standard treatment. Food intake of the patients is reduced after esophagectomy for esophageal cancer. Jejunostomy is performed in patients undergoing esophagectomy to improve their nutrition status in many institutes, and if complications, including leakage and stenosis of anastomosis, recurrent nerve palsy, pneumonia etc, occur after esophagectomy, the patient cannot take anything by mouth over the long-term. In such cases, jejunostomy tube placement is performed in order to improve their nutrition status and QOL. We developed the SLJT placement to improve nutrition in patients undergoing esophagectomy since March 2008. The enteral route is considered more physiologic - the liver is not bypassed and the hepatic ability to take up, process, and store the various nutrients for subsequent release upon nervous or hormonal command, is maintained. It is often said that enteral nutrition is more safe and efficacious than the parenteral route[1]. However, previous jejunostomy methods[2,5-7] have utilized a long catheter out of the abdominal wall, which can be inconvenient in daily life. Some authors have reported methods of jejunostomy[2,5-7]. Our jejunostomy utilized that reported by Sano *et al*. [4].

Due to the technical steps required for the creation of a standard jejunostomy, most surgeons resist using this procedure as a routine adjunctive measure[7]. However, our procedure requires less than five minutes for completion. It adds very little time or trauma to the surgical procedure, and intestinal manipulation is not required.

In the supporting nourishment from the jejunostomy tube, a few problems, including the taste of the liquefied standard diets, and pain, as a result of retention of the catheter, could become evident. Furthermore, there has been one report[4] of improvement of a meaningful nourishment state 1 year after total gastrectomy.

Nishi *et al*. reported jejunostomy placement from the Treitz ligament to about 20 cm on the anal side[8]. Our jejunostomy was also performed 20 cm from the Treitz ligament. Sano *et al*. reported one case of leakage of the intestinal juice to the abdominal cavity and that a purse string suture prevented the leakage[4]. We performed a purse string suture for all patients and found that there were no complications of the leakage. However, there were complications, including peristomal leakage, dermatitis, and ulceration, when the SLJT was used for more than three months. Exchange of the G-tube under radiologic guidance was effective in patients with peristomal leakage.

As a rule, we generally remove the G-tube after confirming the amount of postoperative oral meal intake and perform postoperative removal of the G-tube one month later.

Bleeding was observed after removal in some cases. However, the bleeding usually ceased within a short time. The fistula was generally closed within one or two days.

The One major limitation of this study is that it was conducted retrospectively, although the data were recorded contemporaneously. The relatively small number of patients included in this study reflects the prevalence of the disease, but despite this, we have shown that SLJT placement with the G-tube is a safe procedure in patients with EC. Furthermore, the survival benefit from jejunostomy in EC has not previously been proven. Indeed, such a probative study would be difficult to design and control; however, further research is required to evaluate the relationship between nutrition state, morbidity, and mortality in this population.

## Conclusions

This SLJT placement technique using the G-tube is a safe procedure in patients with EC and allows the creation of a long-term feeding jejunostomy. Thus, we emphasize that SLJT is easy, rapid, and has less complications compared with the standard jejunostomy.

## Abbreviations

(SLJT): skin-level jejunostomy tube; (QOL): quality of life; (G-tube): Entristar™ skin-level gastrostomy tube; (PEG): percutaneous endoscopic gastrostomy; (EC): esophageal cancer;

## Competing interests

The authors declare that they have no competing interests.

## Authors' contributions

YR carried out this procedures and wrote the manuscript. NY, HM, TS, KT, TH, TO, NW, MM and TI assisted this procedures. OT and TI participated in paper revise. All authors read and approved the final manuscript.

## Pre-publication history

The pre-publication history for this paper can be accessed here:

http://www.biomedcentral.com/1471-230X/11/8/prepub
